# Dropout Rates in Psychosocial Interventions for People With Both Severe Mental Illness and Substance Misuse: A Systematic Review and Meta-Analysis

**DOI:** 10.3389/fpsyt.2022.842329

**Published:** 2022-05-12

**Authors:** Marianne Bouchard, Tania Lecomte, Briana Cloutier, Jessica Herrera-Roberge, Stéphane Potvin

**Affiliations:** ^1^Department of Psychology, University of Montreal, Montréal, QC, Canada; ^2^Centre de recherche de l'Institut Universitaire en Santé mentale de Montréal, Montreal, QC, Canada; ^3^Department of Psychiatry, University of Montreal, Montreal, QC, Canada

**Keywords:** dropout, psychosocial interventions, severe mental illness, psychotic spectrum disorder, substance use disorder, dual diagnosis

## Abstract

**Introduction:**

Over the years, many psychosocial interventions for individual having both a psychotic spectrum disorder and a substance use disorder diagnoses have been developed and studied. However, there is a high dropout rate among this clinical population.

**Objectives:**

This meta-analysis aims to replicate a previous meta-analysis on the effects of psychosocial treatment for dual disorders, while including and determining the dropout rates in those type of interventions.

**Method:**

Based on a Cochrane systematic review conducted in 2019, we conducted a meta-analysis including 40 randomized clinical trials on psychosocial treatment among persons suffering from schizophrenia spectrum disorder and substance use disorder.

**Results:**

A dropout rate of 27,2% was obtained. Stimulants use significantly affected dropout rates. Age, gender, diagnosis, alcohol and cannabis abuse, and duration of treatment did not affect dropout rates.

**Conclusion:**

The 27,2% rate of dropout from psychosocial treatment highlights the need to engage participants having a dual diagnosis from the start by focusing on therapeutic alliance and motivation for treatment.

## Introduction

Severe mental disorders are defined by the nature of the diagnosis, the degree of disability and the duration of the disorder (1). As such, the following diagnoses are considered severe mental disorders: schizophrenia and related disorders, bipolar disorders, and severe depressive disorders (2–6). Approximately 40–60% of individuals with a serious mental disorder also present with a comorbid substance use disorder (7–9). For individuals with schizophrenia, the risk of comorbid alcohol misuse is three times more likely, whereas the risk for drugs misuse is six times more likely (10), when compared to people without a psychiatric disorder. Overall, people with schizophrenia are 5.3 times at greater risk to present with a substance use disorder than the general population (11). In fact, the proportion of individuals with schizophrenia who present with a substance use disorder is significantly higher than what is found in most other clinical or non-clinical populations (7, 9, 12–14).

It is important to note that severe mental disorders come with a variety of challenges (15), and these are exacerbated with substance misuse, namely isolation, anxiety, depression, suicidal thoughts, behavioral and emotional problems (2). Even mild substance abuse is associated with increased risk for suicide, AIDS, hepatitis, assault, incarceration, homelessness, and fewer social and financial resources (4, 16). Furthermore, substance abuse in severe mental disorders interferes with diagnostic and treatment and causes a multitude of difficulties in a clinical population already facing major difficulties (14, 17).

Over the years, many psychosocial interventions have been developed specifically for this dual diagnosis population. These include interventions and programs such as motivational interviewing (MI), cognitive behavioral therapy (CBT), contingency management (CM), psychoeducation, integrated treatments (IT), psychosocial treatment, and assertive community treatment (4, 18–20). However, people with comorbid severe mental disorders and substance misuse have been described as particularly vulnerable to treatment dropout (21). Ensuring treatment adherence is a major issue in psychiatry, as well as in general medical practice (22). Why are individuals with comorbid severe mental and substance use disorders at higher risk of treatment dropout? In their review on the subject, Kreyenbuhl et al. (23) reported that younger age, male gender, lack of insight, a tendency to minimize symptoms and their impact, and low social functioning as well as a low socioeconomic status was linked to drop out rates. Of the most cited reasons for disengaging is the desire to solve problems on their own (23), dissatisfaction with the treatment or the impression that it wouldn't help, feeling that they already had improved, feeling that they were too unwell, and medication and its side-effects. Other reasons mentioned were having forgotten the appointment and a fear of the mental health system due to previous negative experiences (23). Treatment willingness and engagement can also be influenced by the therapeutic alliance with the therapist, perceived accessibility of care and the client's belief that the treatment will help (24). This is even more an issue for individuals with concurrent substance abuse disorder and/or addiction, with high drop-out rates across treatments (21). Dropping out of psychosocial treatments is associated with a number of clinical, social and economic consequences, as well as higher risk of relapse, re-hospitalization and poorer prognosis. A previous meta-analysis from our team (25) on the drop-out rates from psychosocial treatments among individuals with a psychotic disorder indicated that ~13% (of the 4,374 participants) dropped out prior to, or during, the treatment. Similar dropout rates have been found by Bighelli et al. (26). The authors suggest that these results may be an underestimation of the actual dropout rate due to publication bias in favor of studies presenting lower drop-out rates, as well as the exclusion from the meta-analysis of trials involving patients with psychosis and substance use disorders. This meta-analysis of 74 trials also revealed that drop-out rates were influenced by age, gender, duration of illness, duration of treatment and treatment setting.

Studies that have evaluated the efficacy of psychosocial interventions for comorbid substance misuse disorders have often based their results on the final sample of participants who completed the intervention. These results rarely account for the initial sample approached nor for dropout rates during the study. As a result, high drop-out rates can lessen the statistical power and the generalizability of those studies, and therefore reduce the possibility of detecting significant effects. If calculations of treatment outcomes and success rates are solely based on the small proportion of participants who complete the study, the results could only reflect the outcomes of those who have better prognostic factors and might not be representative of the population of individuals with comorbid severe mental disorder and substance misuse. It is therefore possible that the success rates reported do not represent the treatment reality of individuals with comorbid severe mental illness and substance misuse, given that those with worse prognostic factors will have likely dropped out of the treatment or study.

Recently, Hunt et al. (4) conducted a meta-analysis on the efficacy of existing interventions and programs for comorbid presentation of severe mental disorders and substance misuse. They covered many psychosocial interventions and programs and included 41 trials for a total of 4,024 participants. In sum, the review reported a lack of quality evidence to support any one psychosocial treatment/program over standard care, and they encountered methodological difficulties, which hindered pooling and the interpretation of results. The meta-analysis did not, however, measure drop-out rates, preferring to exclude studies when these rates were too high.

The objective for the present review is to determine the drop-out rates in studies on psychosocial interventions for people with comorbid severe mental disorders and substance misuse (4), for both the experimental and control conditions. As a secondary objective, we will examine the influence of population (e.g., age, gender, diagnosis and substances used) and trial characteristics (e.g., duration of treatment, type of intervention) on drop-out rates.

## Methods

### Eligibility Criteria

This meta-analysis included all RCTs with or without blind randomization which included a comparison between psychosocial intervention aiming at substance abuse reduction and a standard treatment in people with serious mental illness. Quasi-randomized studies were excluded. We opted for RCTs, considering that randomization was our minimal quality criterion, and that our previous meta-analysis on drop-out rates included only RCTs (25). Studies with missing data were excluded. We included participants diagnosed with both a diagnosis of substance misuse and severe mental illness, focusing primarily on psychotic spectrum disorders. Studies that included a vast spectrum of disorders were included only if the majority (e.g., ≥50%) of participants had a diagnosis of severe mental illness. We only included studies published in English or French.

### Data Collection and Literature Search

**Figure 1:**
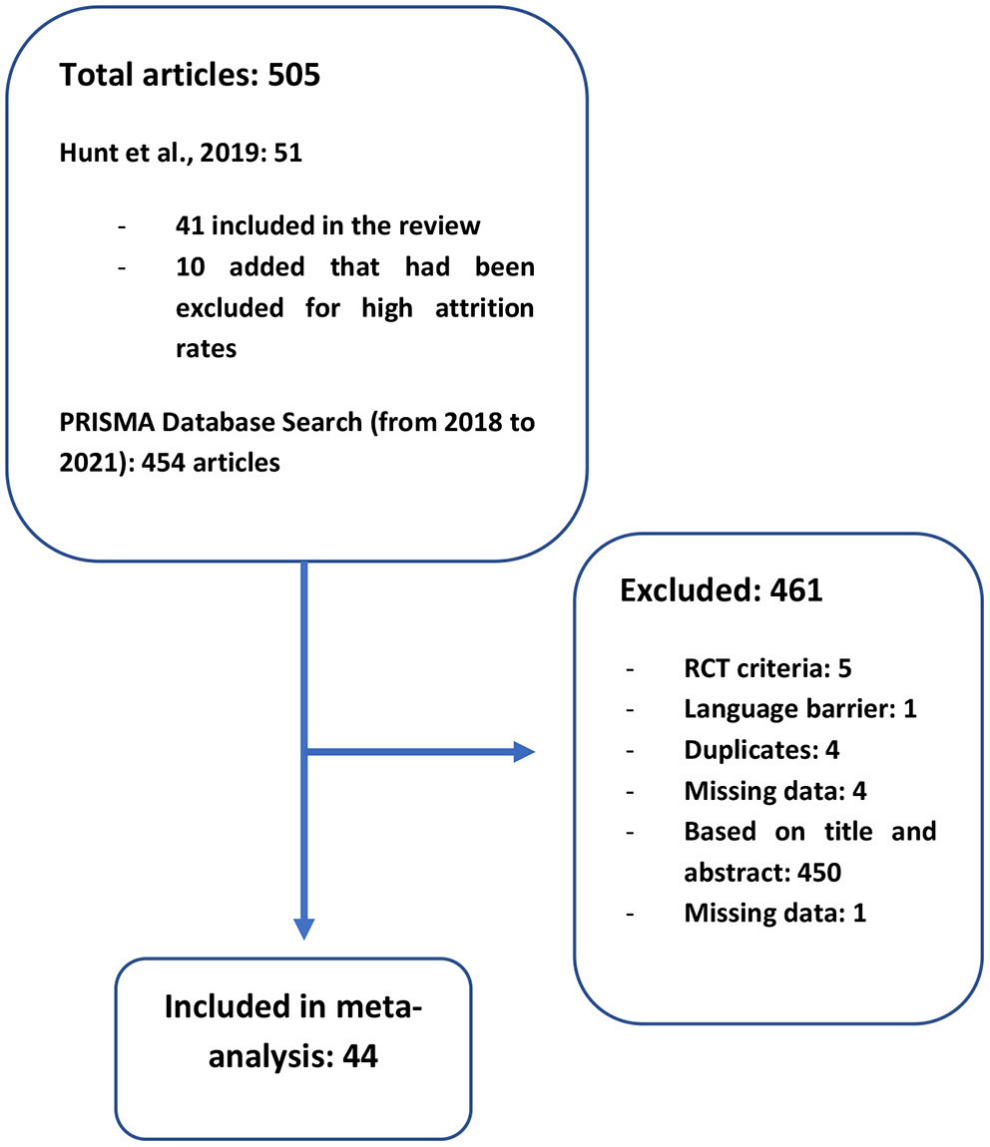
Flow diagram: articles selection process.

We searched Prospero and the existing literature and no meta-analysis on drop-out rates during psychosocial intervention in dual-diagnosis was found. The current meta-analysis included all the articles from Hunt et al. (4) as well as new articles published since. In their Crochrane review, Hunt et al. (4) proceeded to search electronic databases using (^*^{PSY}^*^ in Intervention) AND (^*^Substance Use^*^ in Healthcare Condition) of STUDY in a study-based register that is compiled by systematic searches of majors resources (AMED, BIOSIS, CENTRAL, CINAHL, ClinicalTrials.Gov, Embase, MEDLINE, PsycINFO, PubMed, WHO ICTRP) and their updates. They also searched other resources, such as references lists, journal databases, trials registries, and personal contact. They then proceeded to select the studies by inspecting all citations and identified relevant abstracts, articles, and trials using their inclusion criteria, which have been inspected furthermore to ensure reliability (4). On the 41 articles retained by these authors, 33 were retained in the present article. Of the 8 excluded, 3 were not RCTs, 1 was excluded because of language barrier and 4 were duplicates. Because drop-out rates are the main focus of the present research, we also considered the articles rejected by this Cochrane review and proceeded to recuperate 10 articles that were excluded for high attrition rates by Hunt et al. (4). Two of these 10 articles were excluded because they were not RCTs. We also searched Psychinfo, Embase and PubMed databases using PRISMA criteria for new articles published between 2018 and 2021 using: “Psychotic^*^” OR “psychos^*^” OR “schiz^*^” OR “Severe mental illness” AND “substance use” OR “substance abuse” OR “substance misuse” OR “drug use” OR “Drug abuse” OR “Drug usage” OR “Substance related disorder^*^” OR “drug addiction” AND “Treatment” OR “intervention” OR “psychosocial” OR “program.” We found 454 articles, and 4 new articles were retained based on title, abstract and full-text reads. In sum, we retained a total of 44 articles for analysis. Five other articles have been excluded during data extraction due to missing data. The Flow chart of the selection of studies is shown in Figure 1. Interventions were divided into four categories: Intervention (including CBT, Skills training, MI and CM), Specialized Integrated Services (Integrated treatments for dual disorders), Integrated services with outreach (e.g., assertive community treatment) and Support interventions (e.g., AA). The characteristics of the studies included in the meta-analysis are described in Table 1.

**Table 1:** Details of included studies.

	**Sample characteristics**								**Intervention details**			**Study details**
	**Mean age (interventions)**	**Mean age (controls)**	**% males**	**% psychotic spectrum**	**% cannabis**	**% stimulants**	**% Alcohol**	**N baseline**	**Intervention category** ^*^	**Duration**	**Comparator**	**Study quality (/6)**
Baker et al. (27)	31.71	30.05	75	37	46.80	22.80	60.80	160	Int.	One session (30–45 min)	TAU	4
Baker et al. (28)	28.83	28.83	78.20	86.60	73.10	42	67.30	130	Int.	10 sessions (1 per week)	Routine treatment	4
Barrowclough et al. (29)	31.1	31.1	92	100	61.11	66.67	61.11	36	Integrated	Over 9 months	TAU	4
Barrowclough et al. (30)	37.4	38.3	86.54	100	25.08	NS	47	327	Integrated	Up to 26 sessions over 12 months	TAU	6
Bellack et al. (31)	43.8	41.6	66.40	39.50	1.64	72.10	21.30	175	Integrated	2 times a week over 6 months	Standard care	4
Bogenschutz et al. (32)	42.74	41.09	52.13	18.20	NS	NS	100	121	Support Int.	12 weeks	TAU	2
Bond et al. (33)	31.5	31.5	79	70	NS	NS	61	97	With outreach	18 months	TAU	0
Bonsack et al. (34)	25	25.5	87.10	100	83.70	NS	NS	62	Int.	4–6 sessions over 6 months	TAU	2
Burnam et al. (35)	37	37	84	45	NS	NS	73.46	276	Integrated	3 months	Controls	2
Cather et al. (36)	23.2	23.1	72.50	100	11.20	NS	7.20	404	Integrated	2 years	Usual care	6
Chandler and Spicer (37)	43	43	71.98	54.30	11.70	30.10	31.10	182	Integrated	2.5 years	TAU	2
Drake et al. (38)	32.2	32.2	76.20	100	48.10	14	83	130	With outreach	3 years	TAU	4
Eack et al. (39)	39.68	34.67	71	100	73	NS	81.80	28	Int.	18 months	TAU	4
Edwards et al. (40)	20.9	21.3	72.30	100	48.90	NS	2.20	47	Integrated	3 months weekly sessions	Psychoeducation	4
Essock et al. (41)	36.4	36.6	72	76	NS	NS	73	198	With outreach	3 years	Standard care	2
Gaughran et al. (42)	43.76	44.65	57.64	100	NS	NS	NS	406	Integrated	9 months		6
Gouzoulis-Mayfrank et al. (43)	31.14	30.8	84	100	72	12	12	100	Integrated	18 months	TAU	2
Graeber et al. (44)	42.87	45	96.67	100	86	71	100	30	Int.	One session per week over 3–4 weeks	Educational treatment	2
Graham et al. (45)	39.5	37.69	84.75	71.19	46.70	3.30	40	59	Int.	4 to 7 sessions over 2 weeks	TAU	6
Hellerstein et al. (46)	31.9	31.9	76.60	100	76.60	87.20	91.50	47	Integrated	2 session per week over 8 monts	Non-integrated treatment	2
Herman et al. (47)	33.2	33.2	73.90	28.10	22.70	60.20	73.40	485	Integrated	18 months	standard treatment	2
Hjorthoj et al. (48)	26.6	27.1	75.73	82.52	100	NS	NS	103	Int.	1–2 sessions per week for the first month. and then one weekly over 6 months	TAU	6
Jerrell et al. (49)	NS	NS	NS	NS	NS	NS	NS	98	Integrated	12 months	Standard care	2
Johnson et al. (50)	24	25	86.75	88.36	72	NS	77	551	Int.	12 weeks	TAU	4
Kavanagh et al. (51)	22.6	22.6	60	100	76	24	88	25	Int.	6–9 sessions within 7–10 days.	Standard care	4
Kemp et al. (52)	20.6	20.8	81.25	100	NS	NS	NS	19	Int.	4–6 sessions	TAU	2
Kikkert et al. (53)	45.9	45.9	80.40	81.80	NS	NS	NS	154	Integrated	12 months	TAU	2
Lehman et al. (54)	31	30	74.07	68.52	50	35	79	54	Integrated	12 months	usual community mental health center (CMHC) and psychosocial rehabilitation service	2
Madigan et al. (55)	27.6	28.2	78.41	77.27	100	NS	NS	88	Int.	Once per week for 12 weeks (3 months)	TAU	4
Mangrum et al. (56)	36.5	36.6	49.07	20.93	NS	NS	NS	216	Integrated	12 months	TAU	0
Martino et al. (57)	35.35	35.35	65	51	35	64	82	23	Int.	One session	standard preadmission interview	2
Martino et al. (58)	29.71	34.1	72.70	100	45.80	70.80	41.70	44	Integrated	Two sessions	two-session standard psychiatric interview	2
McDonell et al. (59)	43.01	42.45	65.34	39.20	NS	96	47	176	Int.	3 months	TAU	2
McDonell et al. (60)	44.55	46.23	63.29	30.38	NS	NS	100	79	Int.	12 weeks	Noncontingent control group (reinforcers regardless of EtG results and treatment attendance)	2
Morse et al. (61)	40	40	80	80	19	NS	82	149	With outreach	24 months	Standard care	2
Mowbray et al. (62)	33.4	33.4	74	28	NS	21.67	NS	467	Integrated	Minimum 28 day stay in the ward	standard inpatient psychiatric treatment	2
Naeem et al. (63)	40.47	40.47	77.01	100	NS	NS	NS	105	Int.	6 sessions over 3 months	Standard care	4
Nagel et al. (64)	33.4 & 32.2	33	57	49	65	NS	63	49	Int.	From 2 to 6 months	Standard care	2
O'Connell et al. (65)	37.7 & 36.8	30.1	66	100	NS	NS	NS	137	Int.	3 months	Standard care	2
Petry et al. (66)	41.7	41.7	58	16	15.80	100	36.80	19	Int.	8 weeks	TAU	2
Rosenblum et al. (67)	42	44	68	30	NS	NS	NS	349	Support Int.	3–6 months	Waiting list control group	2
Swanson et al. (68)	32.85	34.87	63.63	44.63	NS	NS	NS	93	Int.	15 minutes of feedback and a 1 h session	Standard care	2
Tracy et al. (69)	NS	NS	50	NS	NS	NS	NS	30	Int.	4 weeks	Assessment only	2
Xie et al. (70)	32.4	32.4	77.60	100	45	15.20	82.70	223	Integrated	3 years		4

* *TAU, treatment as usual; Int., Intervention (e.g. CBT, contingency management, motivational interviewing). Integrated: Specialized integrated services; With outreach, integrated community treatment with outreach (e.g. assertive community treatment); Support Int, support interventions (e.g., 12–steps)*.

### Data Extraction and Quantitative Data Synthesis

For drop-out rates, the number of participants suffering from a severe psychiatric disorder prior to treatment and at the end of treatment, respectively, was extracted from each study. Data on age (average age in terms of years), sex ratio (percentage of males and females), duration of treatment (number of weeks), treatment modality (interventions such MI and/or CBT, specialized integrated services, intervention with outreach, and support intervention), and percentage of patients with alcohol, cannabis and stimulant use disorders were also gathered. Data extraction was verified by two authors of this article. The *Comprehensive Meta-Analysis*-2 software (71) was used to conduct analyses of effect size, which corresponds to the drop-out rate (e.g., event rate), which represents the loss of participants prior or during treatment among those who agreed to undergo the treatment. Heterogeneity among effect size estimates was assessed with the Q statistics (72), with magnitude of heterogeneity being evaluated with the I^2^ index (73). As the database was characterized by high heterogeneity (see below), we aggregated event rates across studies using random-effects models, which are more conservative than fixed-effect models, and seem to better address heterogeneity between studies and study populations (74). The possibility of publication bias was examined with Egger's test and visual inspection of funnel plot (75). Sub-analyses were conducted on treatment modality (e.g., intervention, specialized integrated services, integrated services with outreach and support interventions). Meta-regression analyses were used to examine the effects on drop-out rates of continuous variables, namely age, sex ratio, percentage of psychotic patients, duration of treatment, study quality and percentage of specific SUDs (e.g., alcohol, cannabis and stimulants). Finally, using event rates as the effect size, we calculated consent rates, which represent the number of patients who consented to participate in the study relative to those who were approached by the research team.

### Data Analysis

Hunt et al. (4) appraised study quality, and evidence was rated as low or very low quality. They report a high or unclear risk of bias because of poor or inadequately reported trial methods, imprecision due to sample sizes, low event rates and wide confidence intervals. We also assessed study quality for the RTCs that were retained for the present article, using Jadad criteria (76). Random allocation, allocation concealment and blindness were the three criteria used, and we adapted the scale for the present research by excluding poor ratings for withdrawals and drop-outs because it was what interested us for the present study. To ensure validity, we conducted two quality evaluation by two researchers to validate and verify Jadad scores. Studies were of low to moderate quality, primarily because of missing data and absence of allocation concealment. Blindness was also not reported or described in a large proportion of the studies included. Study quality for each trial, as determined using Jadad criteria, is detailed in Table 1.

## Results

### Drop-Out Rates

**Figure 2:**
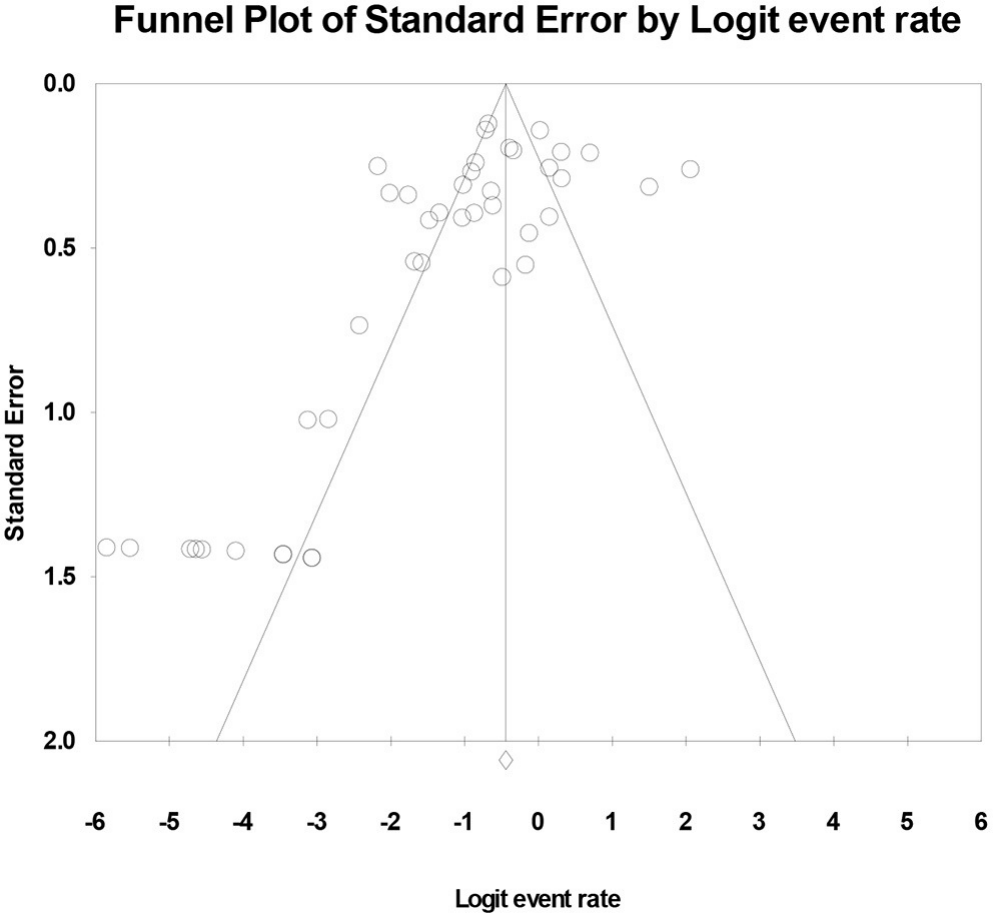
Publication bias for the composite analysis on experimental treatments.

In the 42 treatment arms, the composite drop-out rate was 27.2% (CI, 95%: 21.0–34.3%) (Table 2). In the case of treatment-as-usual (TAU), the aggregation of 32 studies produced a composite drop-out rate of 20.5% (CI, 95%: 14.2–28.6%) (Table 2). As illustrated in Figure 2, a publication bias was present (Kendall's Tau = −0.309; *p* = 0.004; Egger's test: *t* = 3.197; *p* = 0.003). For both experimental treatment and TAU, results across treatment arms were characterized by very high levels of heterogeneity (I^2^ = 90% and 90.1%, respectively) (Table 2).

**Table 2:** Primary and secondary analyses: drop-out rates across interventions.

**Analysis**	**Number of treatment arms**	**Rate (%)**	***p*** **-value**	**Confidence interval**	**Heterogeneity**
Main analysis					
Experimental treatment	42	27.2	0.0001	(21.0–34.3)	Q = 409.3; *p* = 0.0001; I^2^ = 90%
TAU	32	20.5	0.0001	(14.2–28.6)	Q = 312.5; *p* = 0.0001; I^2^ = 90.1%
Sub-analyses (for experimental treatment arms only)					
Intervention ^*^	20	28.7	0.0001	(19.5–40.2)	Q = 79.2; *p* = 0.0001; I^2^ = 76%
Specialized Integrated Service	16	27.3	0.001	(17.3–40.3)	Q = 269.7; *p* = 0.0001; I^2^ = 94.4%
Outreach	4	11.1	0.002	(3.3–31.5)	Q = 14.5; *p* = 0.002; I^2^ = 79.4%
Support therapy	2	28.3	0.209	(8.4–62.8)	Q = 30.6; *p* = 0.0001; I^2^ = 96.7%

*TAU, treatment-as-usual*;

* *Intervention, motivational interviewing and/or cognitive behavioral therapy*.

### Secondary Analyses

A sub-analysis on treatment modality showed that drop-out rates were fairly similar across interventions (28.7%; 20 treatment arms), specialized integrated services (27.3%; 16 treatment arms) and support therapies (28.3%; 2 treatment arms), but that drop-out rates were lower in trials on interventions with outreach (11.1%; 4 treatment arms) (Table 2). Within each treatment modality, results were characterized by high levels of heterogeneity (between 76 and 96.7%).

**Figure 3:**
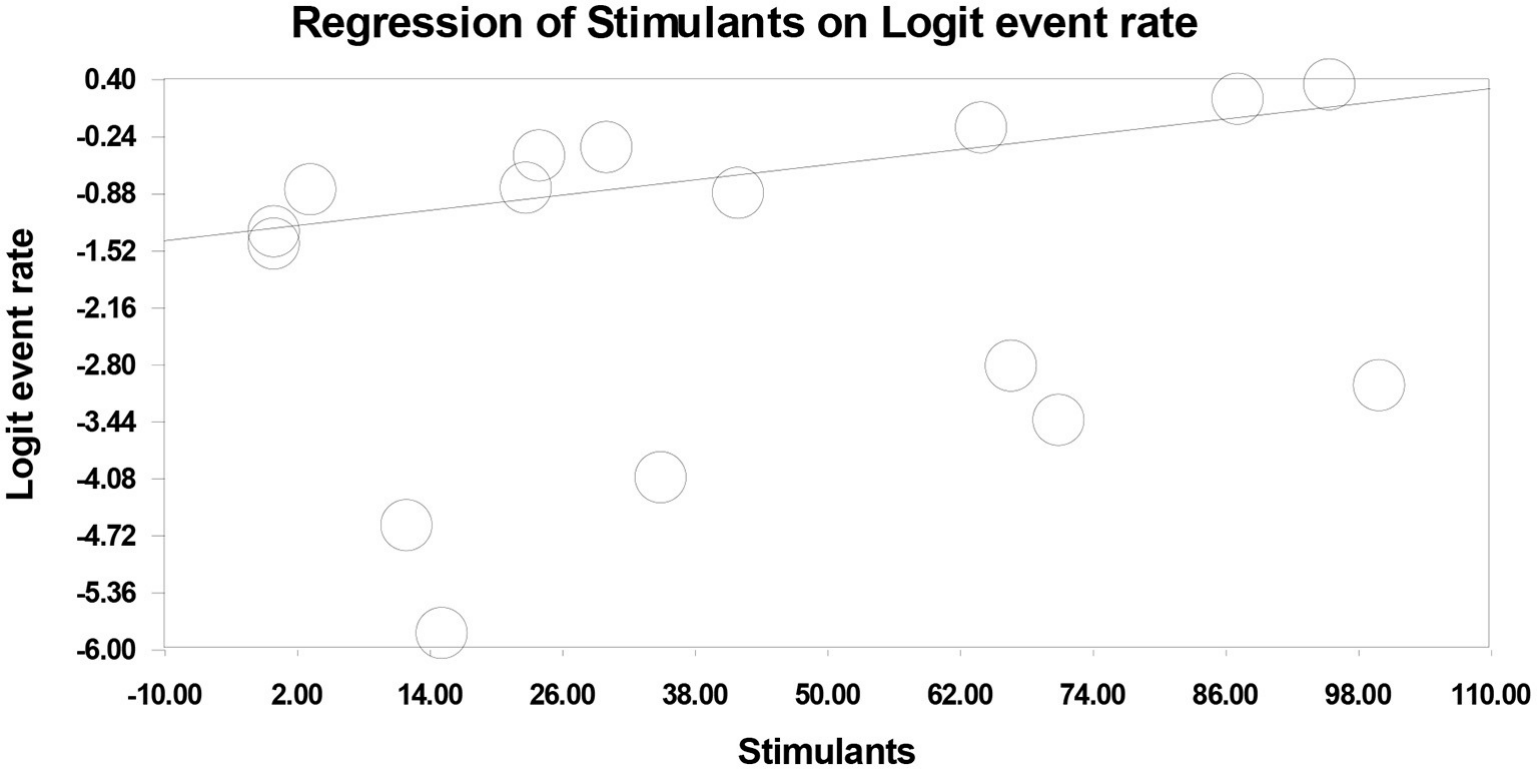
The influence of percentage of patients with stimulant use disorder on drop-out rates across experimental treatments.

Meta-regression analyses on the experimental treatment arms showed a positive association between stimulant use disorder (StUD) and drop-out rates [16 experimental treatment arms; slope (β) = 0.014; *p* = 0.0001] (Table 3; Figure 3). That is, the highest drop-out rates were observed in trials including the highest proportion of patients with as StUD. Conversely, age (*p* = 0.530), sex ratio (*p* = 0.561), percentage of psychotic patients (*p* = 0.119), duration of treatment (*p* = 0.129), study quality (*p* = 0.967), percentage of patients with alcohol use disorder (*p* = 0.464) and percentage of patients with cannabis use disorder (*p* = 0.091) had no significant influence on drop-out rates across trials (Table 3).

**Table 3:** Predictors of drop-out rates for experimental treatments.

**Predictor**	**Number of experimental treatment arms**	**Slope**
Age	39	β = 0.021; *p* = 0.530
Sex ratio	41	β = 0.012; *p* = 0.561
Duration of treatment (in weeks)	40	β = −0.010; *p* = 0.129
% of patients with psychosis	40	β = −0.013; *p* = 0.119
% of patients with alcohol use disorder	30	β = 0.008; *p* = 0.464
% of patients with cannabis use disorder	27	β = 0.015; *p* = 0.091
% of patients with stimulant use disorder	16	β = 0.014; *p* = 0.0001
Study quality	42	β = −0.006; *p* = 0.967

### Consent Rates

In the 29 studies offering this information, we found that the composite consent rate was 44.4% (CI, 95%: 0.365–0.526; *p* = 0.178). Across studies, results were highly heterogeneous (Q = 2,088.2; *p* = 0.0001; I^2^ = 98.7%).

## Discussion

The objectives of the present research were to determine dropout rates in studies on psychosocial interventions for people with a dual diagnosis of severe mental illness and substance abuse. We also wanted to examine the influence of population (e.g., age, gender, diagnosis and substances used) and trial characteristics (e.g., duration of treatment, type of intervention) on drop-out rates.

The dropout rate of 27.2% for the experimental arm and of 20.5% for TAU suggest that, on average, close to one third of participants in treatment studies never complete the treatment. Furthermore, the publication bias found suggest that studies under report their drop-out rates, which brings us to suppose that the actual drop-out rates might be even higher. Dropout rates results had high heterogeneity (I^2^ ranging from 76 to 96,7%). This variability might be explained by publication bias, high differences in outcomes measured in studies, and differing ways in which psychosocial interventions were delivered.

Villeneuve et al. (25) found a dropout rate of 13% in their meta-analysis on dropout from psychosocial treatment among individuals with schizophrenia spectrum disorder, which is less than half of what we found in our experimental arm. One of the major differences between their study and ours is that we included persons with both schizophrenia spectrum disorder and substance use disorder. Differences in dropout rates could partly be explained by stimulants use, since the dropout rates appeared worse in those with stimulant use. This difference in results could also possibly be explained by higher severity of symptoms, impulsivity, and lower motivation in our clinical population, although these were not specifically analyzed here. Individuals with a dual diagnosis of psychotic spectrum disorder and substance use disorder in general present with higher symptom severity and more relapses, as well as more deficits in executive functions like planning and thinking before acting, more impulsivity and less motivation in general (77, 78). Another factor that could explain the drop-out rates result is a poor therapeutic alliance (23, 24), and demanding requirements for certain interventions (for example, many interventions required participants to come to clinics regularly, in person) even though motivation is often an issue with this population.

Research on dual diagnoses focus on developing and replicating studies on specialized treatments to demonstrate their efficacity. However, these different interventions and treatment programs present few similarities and vary greatly on the type of interventions they include, the objectives, and the symptoms targeted (79). This brings us to wonder if the research focus should shift away from a focus on the efficacy of specialized treatments, since there is a high dropout rate form experimental treatments and, therefore, the results might only represent the small portion that accept to participate and complete the treatment (25).

Our results also showed a lower drop-out rate, of 11,1%, for intensive programs like assertive community treatments. Although this rate was based on the aggregation of only 4 trials, it is noteworthy that this rate was half of the rate found in more specific treatments in this review. Such programs focus on engaging the participant, are long-term, and do not specifically aim on obtaining results regarding substance misuse (80, 81). In their meta-analysis, Hunt et al. (4) found no difference between treatments in terms of improved outcomes in terms of lost to treatment, death, alcohol or substance used, global functioning and general satisfaction, suggesting that intensive treatment programs like assertive community treatments did not fare better or worse than the other treatments or services analyzed. Most studies search for gains in terms of outcomes (decreased substance use, improved symptoms and global functioning for example), yet, with complex clinical populations such as people with comorbid substance misuse and psychotic disorders, the evolution in terms of clinical outcomes can be slow, suggesting a need for long-term treatments that focus on engaging the person and developing a strong therapeutic alliance. It is also important to consider the complexity of this clinical population. There is often history of abuse and trauma (82, 83), emotional self-regulation issues (84), frequent comorbidity with personality disorders (85), and with anxiety disorders (86), and important cognitive and functional deficits. There is also medication to consider, which can lead to a multitude of side effects depending on dose and type of medication, that can interact with substance misuse. These issues can be a challenge to work with since there are many parameters to account for, and can make it difficult to develop a strong and good therapeutic alliance both from the client and the clinician's perspective (87–89). Interventions should perhaps focus more on engaging participants by developing a strong therapeutic alliance, with the hope of eventually motivating them in working on their substance misuse problem. As discussed, many reasons for dropout reported by participants in studies were related to engagement with the therapist or team (23, 24). This suggests that a more engaging approach to treatment, with outreach such as in assertive community treatment teams, might be more successful in the long run in keeping clients into treatment. Having intensive treatment teams trained to work with psychotic spectrum and substance use disorders (and more specifically in stimulant use disorders) while targeting the development of a good alliance with the participant appears promising to prevent dropout rates.

Although we did not find a significant association between study quality and drop-out rates, it must be pointed that studies included in the current meta-analysis were in vast majority of low to moderate quality. This was mostly due to the absence of allocation concealment most trials and due to the fact that blindness was also not reported or described in most cases. At face value, this may seem to be a limitation, as there were few high quality to analyze. However, one may argue that the access to low / moderate quality trials may be better suited for the assessment of drop-out rates. This can be explained by the fact that higher quality studies often have more resources at their disposition to conduct their research, and thus are more able to invest in research teams and labs that can either follow the participants more closely or pay them more for their participation. Thus, higher the quality trials may, in theory, be more biased in the assessment of dropouts. On the other hand, low quality studies might in fact be more accurate in assessing the clinical reality of offering interventions to people with a severe mental illness and concurrent substance abuse. As such, having more low-quality studies in the context of this research topic is advised as these are perhaps more ecologically valid than high quality studies. In the future, it will be important to collect, in a systematic manner, data on drop-out rates during psychosocial interventions delivered in non-randomized trials. Although, as described by Hunt et al. (4), treatment outcomes are not impressive with this population, future studies should also investigate how drop-out rates affect actual treatment effect sizes.

## Data Availability Statement

The original contributions presented in the study are included in the article/supplementary material, further inquiries can be directed to the corresponding author.

## Author Contributions

MB conducted the review and wrote the article. TL co-supervised MB, edited the article. BC conducted the interrater agreements. JH-R formatted the article, references and helped with the data search, and SP co-supervised MB and conducted the analyses. All authors contributed to the article and approved the submitted version.

## Conflict of Interest

The authors declare that the research was conducted in the absence of any commercial or financial relationships that could be construed as a potential conflict of interest.

## Publisher's Note

All claims expressed in this article are solely those of the authors and do not necessarily represent those of their affiliated organizations, or those of the publisher, the editors and the reviewers. Any product that may be evaluated in this article, or claim that may be made by its manufacturer, is not guaranteed or endorsed by the publisher.
